# Navigating Uncertainty: Teachers’ Insights on Their Preservice Training and Its Influence on Self-Efficacy during the COVID-19 Pandemic

**DOI:** 10.3390/bs14020135

**Published:** 2024-02-13

**Authors:** Yonit Nissim, Eitan Simon

**Affiliations:** Department of Education and Learning, Tel Hai College, Qiryat Shemona 1220800, Israel; eitans@telhai.ac.il

**Keywords:** teachers’ self-efficacy, retrospective perception, COVID-19

## Abstract

This quantitative study investigates teachers’ perceptions of self-efficacy during the COVID-19 pandemic and explores the correlation between these perceptions and the preservice training they received. The research addresses the cognitive connection between teachers’ current self-efficacy, particularly their satisfaction with and appreciation of preservice lecturers. The connection between self-efficacy and “cognitive connection” lies in the intricate interplay of cognitive processes, observational learning, and the formation of beliefs and perceptions. The way individuals cognitively process information, make connections between experiences, and interpret feedback significantly influences their self-efficacy beliefs and behaviors. Utilizing a retrospective lens, the study reveals a significant correlation between teachers’ evaluation of their preservice training, especially their appreciation of lecturers, and their present self-efficacy. The findings highlight that teachers, amidst the challenges of the pandemic, evaluated their self-efficacy at a remarkably high level. This underscores their resilience during a period of unprecedented uncertainty demanding substantial personal and professional adaptability. The nuanced interplay observed suggests that teachers’ sense of self-efficacy serves as a predictive variable of their mental and professional resilience when confronting uncertainty and navigating rapid and profound changes, as exemplified by the exigencies of the COVID-19 pandemic.

## 1. Introduction

A plethora of research has investigated the performance and functioning of teachers amid the COVID-19 crisis globally and within the specific context of Israel [1,2]. Schön’s concept of reflective practice (1983) informs the study’s approach to understanding the impact of prior training on teachers’ adaptability and resilience in crises. However, a research gap persists regarding teachers’ sense of self-efficacy in the face of the intricate situations arising from the pandemic and the contributing factors influencing this sense [3]. This current study aims to address this gap by adopting a research approach derived from retrospective observation [4,5]. This approach mediates by providing a layered depiction of the evolving sense of self-efficacy tested in times of crisis [6]. It allows for a deeper understanding of the importance of optimal preservice training and its lasting effects on teachers’ abilities to adapt and succeed in challenging situations, such as online teaching during a crisis.

The study addresses the gap by exploring the significance of prior teacher training in evaluating self-efficacy during the pandemic. It aims to construct a comprehensive framework that reflects teachers’ perceptions of their training and explores the enduring impact on self-efficacy and professional resilience during crises. Self-efficacy in online teaching differs from that in traditional classrooms due to the unique demands of the online environment. Teachers must navigate technology, maintain student engagement remotely, and address challenges specific to the online setting.

The concept of self-efficacy is crucial in the context of teachers and teaching. Teachers with higher self-efficacy demonstrate greater enthusiasm, openness to new ideas, willingness to experiment with new methods, and a stronger commitment to teaching. These characteristics are positively linked to a teacher’s likelihood to stay in the profession, as supported by various studies [7,8,9,10].

Studying teachers’ reflections on their training is crucial for understanding the impact on their self-efficacy [10]. Positive experiences during teaching practice enhance preservice teachers’ confidence. A supportive teacher community fosters beliefs and self-efficacy, influencing novice teachers’ well-being and reducing attrition. Examining teachers’ retrospective views empowers them by identifying factors contributing to their self-efficacy and success in the profession [11].

Studying teachers’ self-efficacy during the pandemic is crucial due to unique challenges in transitioning to online teaching. In this complex situation, self-efficacy differs from traditional settings, as teachers face unfamiliar tools and uncertainties. Their confidence and adaptability are essential for successfully navigating these challenges. Understanding their preparedness offers insights for targeted professional development. The impact of the pandemic on teacher self-efficacy varies, with some facing challenges but many demonstrating resilience. The study explores the significance of prior teacher training in evaluating self-efficacy during the pandemic. It aims to construct a comprehensive framework, reflecting teachers’ inner world and perceptions of training and providing insight into the enduring impact on self-efficacy and professional resilience during crises. The retrospective observation acts as a mediator, offering a layered depiction of the evolving sense of self-efficacy tested in times of crisis. Studying the correlation between past training and the sense of self-efficacy in the present is integral for refining training practices, supporting teacher well-being, and ultimately improving the overall quality of education.

### 1.1. Literature Review

In recent years, scholarly interest in teachers’ self-efficacy has grown, recognized for its significance in fostering efficiency and satisfaction, reducing burnout rates, and preventing dropout from the teaching profession [12]. Contemporary studies underscore the positive impact of enhancing teachers’ expertise, professional development, collaborative efforts, and teaching quality on their sense of efficacy [13]. Teacher self-efficacy (TSE), representing a teacher’s confidence in various professional activities, significantly influences classroom processes and student academic adjustment. Extensive research has explored TSE’s connection to teachers’ well-being, including burnout, stress, coping mechanisms, job satisfaction, and professional commitment. Various studies have consistently highlighted the importance of TSE in shaping both teaching dynamics and teachers’ overall job satisfaction and mental well-being [14]. 

### 1.2. Teacher Education in Israel

The Israeli teacher education model spans four years across 21 colleges and 9 universities, culminating in a B.Ed degree and teaching certificate for preservice teachers (PSTs). This holistic program integrates education and discipline-specific courses with practical experiences, embodying a “learning by doing” ethos [15,16]. Evaluations of teacher education extend to teacher quality, teaching efficacy, and impact on student achievements [17]. Teacher education institutions emphasize the pivotal role of teacher educators, lecturers, and counselors as PST role models, shaping PSTs’ professional identities [18].

Recent findings reveal positive correlations between graduates of teaching programs, collaborative models, high self-efficacy, and readiness for teaching [19,20]. Notably, heightened self-efficacy predicts novice teachers’ commitment beyond three years in the profession [21]. Despite this, approximately 45% of novice teachers in Israel leave the education system within five years (data presentation to presidents of colleges of education and teaching, 2021), underscoring the practical importance of studying teachers’ self-efficacy and its influencing factors.

### 1.3. Teachers’ Self-Efficacy

Educators’ self-efficacy in online teaching is often diminished due to the notable disparities between physical and digital classrooms [22]. These distinctions raise concerns about teachers’ perceived effectiveness and competence in navigating online instructional methodologies. Teachers often experience lower self-efficacy in online teaching due to the differences between physical and online classroom environments [22,23]. Notably, educators who have previous experience in online teaching tend to express higher motivation for teaching in the online setting.

Teacher self-efficacy beliefs have been a topic of great interest because of their potential to positively influence teacher actions and student outcomes [2,24]. The concept of teacher self-efficacy is grounded in the experience of control, reflecting their perceived ability to succeed in teaching tasks [14]. High self-efficacy correlates with greater resilience, job satisfaction, well-being, and occupational commitment [25,26]. Teachers’ self-efficacy is crucial to teaching quality and student support and even predicts teachers’ commitment to the profession. The COVID-19 pandemic has added a new layer of challenges, requiring teachers to adapt quickly and flexibly, thus influencing their perceptions of self-efficacy [27].

#### Preservice Teachers’ (PSTs’) Self-Efficacy

Preservice teachers’ self-efficacy, aligned with social learning theory, is cultivated through essential field experiences like practicum and student teaching. These opportunities, emphasizing observation, practice, and feedback, are integral to teacher preparation programs and enhance teaching skills [28]. State mandates ensure preservice teachers spend supervised time in K-12 schools, culminating in a comprehensive “student teaching” experience where they assume full teaching responsibilities. It is also believed that the impact on student achievement [29] evolves during preservice training, particularly through student teaching. 

Notably, research indicates a significant increase in self-efficacy during the student teaching period, underscoring the importance of observation and practice in skill development [30].

From a social cognitive perspective, self-efficacy, defined as individuals’ belief in their ability to accomplish daily tasks, significantly influences decision-making processes. High self-efficacy is associated with setting challenging goals, enhanced resilience, and reduced negative emotions during goal attainment [31]. Research, particularly in teacher education, emphasizes the pivotal role of teacher self-efficacy (TSE) in shaping personal goals, perseverance in the face of challenges, and motivation for specific teaching behaviors, including the use of digital teaching materials [32] Higher TSE correlates with increased engagement, job satisfaction, and persistence in handling teaching adversities, leading to more creative teaching strategies [33]. Additionally, it is linked to teacher retention at both preservice and in-service levels and impacts preservice teachers’ lifelong learning competencies [34]. 

Although existing studies on TSE predominantly focus on physical classroom teaching, the Teachers’ Sense of Efficacy Scale (TSES) is a widely used tool covering instructional strategies, student engagement, and classroom management [7]. To encompass broader teaching domains, the Norwegian Teacher Self-efficacy Scale introduced six dimensions, including instruction, adapting education to individual needs, motivating students, discipline management, collaboration with colleagues and parents, and coping with changes and challenges [35]. Adaptations of TSES for preservice teachers (PSTs) reveal a stable three-dimensional structure, emphasizing the need for specific scales aligned with the unique challenges of different teaching contexts [36]. 

Although efforts have been made to adapt TSE scales to measure domain-specific efficacy, such as literacy skills, these adaptations have primarily been explored in physical classroom teaching, leaving a gap in understanding self-efficacy in online teaching contexts [37]. A study by Robinia (2008) adapted TSES for online teaching, revealing a validated two-factor structure encompassing TSE for online instruction and online technology, highlighting the need for further exploration in the realm of online teacher self-efficacy [38]. 

The literature gap in teachers’ self-efficacy refers to areas within research that have not been adequately explored or studied. Specifically, in the context of online teaching, there are several aspects that researchers have not sufficiently addressed:

1. Online Teaching Context:

Existing studies have primarily focused on teachers’ self-efficacy in traditional, face-to-face classrooms. The gap lies in the lack of research specific to the challenges and dynamics of online teaching.

Technological Competence:

With the increasing use of technology in education, there is a need to understand how teachers’ confidence in their abilities (self-efficacy) is connected to their proficiency in using digital tools for online instruction.

2. Adaptability and Innovation: Little research has been conducted on how teachers’ self-efficacy influences their ability to adapt to and innovate within the unique setting of virtual classrooms.
3. Professional Development Needs: The literature gap includes a lack of exploration into the specific training and development needs related to teachers’ self-efficacy in the context of online teaching.
4. Student Engagement: Understanding how teachers’ self-efficacy impacts their interactions with and engagement of students in online settings is an area that has not been thoroughly investigated.
5. Long-Term Effects: There is a scarcity of longitudinal studies examining how teachers’ self-efficacy evolves and persists over time in the context of online teaching. Addressing these gaps under the circumstances of the COVID-19 pandemic is crucial for a more complete understanding of how teachers’ confidence and belief in their abilities influence their effectiveness in online education. It can also inform the development of targeted training programs and policies to support teachers in adapting to the changing landscape of modern education.

## 2. Methodology

This research adopts a quantitative approach employing a self-report questionnaire to investigate retrospective reflections on prior training experiences. By focusing on participants’ memories, this method aims to unravel the past processes that continue to influence the present. The study’s robustness and reliability are fortified through the triangulation of information sources, a comprehensive literature review, and the use of a validated questionnaire. We identified the key themes from the literature review, used the survey data findings to identify commonalities or discrepancies, and relied on the theoretical frameworks to interpret the survey results. We identified the themes that emerged in both the literature and the survey responses to strengthen the validity of our conclusions.

This instrument, originally established and subsequently adapted for the study’s specific objectives, underwent a meticulous face validation process: We assembled a panel of three experts to review and provide feedback on the survey. The survey was revised based on expert feedback. A pilot test was conducted by three content experts with doctorates in education with a small group to identify any issues with clarity or understanding. Following individual scrutiny and collaborative discussion, the questionnaire underwent a pilot phase involving 50 participants to assess its validity and reliability, yielding a high Cronbach’s alpha reliability coefficient (α = 0.922). The confirmed validity and reliability ensured the subsequent distribution of the questionnaire to participants in subsequent study stages, underscoring the methodological rigor employed to facilitate a thorough examination of the study’s objectives.

### 2.1. Research Tools

The research tool was a validated anonymous self-report questionnaire with four parts:

1. Demographic details—background, age, education, socioeconomic status, socio-religious affiliation, and teaching seniority, plus two more sections with closed questions. The questionnaire consisted of two research questionnaires recognized in the research literature as follows:
2. An evaluation and satisfaction questionnaire (based on Ayllón et al. 2019) [39] involving retrospection on satisfaction with their lecturers and preservice training. This section contained 7 statements. Statements 1 and 4 expressed general appreciation for the structure of the preservice training; statements 2 and 5 expressed the teachers’ general perceptions of their self-efficacy. Statement 3 expressed the lecturers’ autonomous support for the PSTs. Statement 6 expressed the lecturers’ support and personal attention, and statement 7 expressed general satisfaction with the preservice training. Table 1 below presents the general characteristics of these statements. The reliability of the statements, as tested according to Cronbach’s alpha, was found to be high (α = 0.922).
3. The General Self-Efficacy Questionnaire (GSE) is based on Chen and Gully (1997) [40], and the version revised by Chen, Gully, and Eden (2001) [41] is based on the Overall Self-Efficacy Test (GSE). This section included 8 statements. Table 1 below presents the general characteristics of these statements.

### 2.2. Distribution and Data Collection

The questionnaire was distributed in a single phase to a convenience sample of some 800 teachers who were graduates of a northern Israeli college of education and had completed their studies in the previous decade. The questions were preceded by an explanation of the research aims and the respondents were promised strict anonymity. The questionnaire was distributed via the college email system at the height of the COVID-19 pandemic and remained available for three months, until March 2021. This was a period when teachers were required to excel in light of the long lockdowns. Data processing included accepted descriptive statistical tests to obtain as accurate a picture as possible with SPSS software (mean, SD, and two-variable Pearson moment) and *t*-tests.

### 2.3. Research Questions

1. Is there any correlation between past training and the sense of self-efficacy in the present?
2. How do teachers rate their self-efficacy during the studied period of the pandemic?
3. Which factors influence how teachers rate their self-efficacy? Which factors’ influences were not proven?

### 2.4. Research Population

The participants included 165 teachers from northern Israel working in the education system who had completed their teacher education in the previous 10 years. Of these, 115 (69.7%) were women and 50 (30.3%) were men. The age range was 25–60 (M = 37.96; SD = 8.04), with seniority ranging from 0 to 31 years (M = 7.98; SD = 6.59). Most participants were married (81.2%) and Jewish (72%), and nearly all were academics (98.8%). Table 2 below presents the distribution of the participants according to demographics: 

Results:

Table 1 presents the teachers’ responses for the evaluation of their preservice training lecturers at the college.

The validity of measures for structure and self-efficacy, as examined via Cronbach’s α, was high, indicating a high level of stability and consistency in the responses of the participants for the statements of each measure. Table 1 displays the teachers’ responses to the various statements about their self-efficacy. 

The perception of self-efficacy was evidently high for all the general statements examined, with determination ranking highest (4.40). This indicates perseverance, endurance, and very high perception of self-efficacy on the one hand, and on the other, the high score for all measures indicates teachers’ overestimation of their self-efficacy, which is impressive, given the complexity of the pandemic period. 

### 2.5. Inferential Statistics 

To examine correlations between the evaluation of the lecturers and overall self-efficacy, Pearson tests were conducted for each of the five measures of lecturer evaluation and the eight statements about self-efficacy. The results are shown in Table 3. 

Most coefficients were significant and positive, so we can say that the more the teachers appreciated their preservice training (and their lecturers), the higher their sense of self-efficacy. For gender differences in lecturer evaluation and overall self-efficacy, *t*-tests were performed for independent samples. Table 4 presents the averages for both groups and the test results. 

As Table 4 shows, no significant differences were found for gender in either the measures of lecturer evaluation or the statements of overall self-efficacy. Hence, gender had no significant impact on the teachers’ perception of their professional self-efficacy or of their preservice training.

To examine correlations between age and seniority and the evaluation of lecturers and overall self-efficacy, Pearson tests were performed. The results are presented in Table 5 below.

A positive correlation of weak but significant intensity was found between age and the measures of self-efficacy and autonomous support in the lecturers’ evaluation: The older the teacher, the higher the levels of self-efficacy and autonomous support. Positive correlations of weak intensity between age and the statement, “I can succeed in any educational task when I am determined,” were also found, and positive correlations of weak intensity were found between seniority and most statements of overall self-efficacy. *t*-tests were performed for additional independent samples to examine differences between teachers with a master’s degree and those with a bachelor’s degree or teaching certificate only for the evaluation of lecturers and overall self-efficacy. Table 6 below presents the averages between the two groups and the results of the tests.

As shown above, teachers with a master’s degree reported that the lecturers presented the curriculum and evaluation criteria clearly and that the course support materials provided by the lecturers helped them (structure index) more positively compared to teachers with a bachelor’s degree. Also, those with a master’s degree reported more autonomous support and lecturer involvement than those with a bachelor’s degree. Likewise, in all aspects of self-efficacy, there were (mostly significantly) higher averages among teachers with a master’s degree than teachers with a bachelor’s degree. Thus, the more educated the teacher, the greater the perception of self-efficacy. The training is in-depth and optimal, affording a sense of empowerment. For differences based on family status (married/single) vis-à-vis lecturer evaluation and overall self-efficacy, *t*-tests were performed for additional independent samples. Table 7 below presents the averages among the two groups and the results of the tests.

Table 7 shows that married teachers reported that they can achieve the educational goals they set for themselves to a significantly greater extent than single teachers. Likewise, married teachers felt that they can perform the educational tasks they face significantly better than their unmarried counterparts. In the other aspects of overall self-efficacy, the average scores for married teachers were also higher than those of single teachers but not significantly. In most of the lecturer evaluation indices, the average scores for married teachers were higher than those for single teachers (with the exception of the overall evaluation) but not significantly.

Summary of the findings:

The empirical findings evince a noteworthy correlation between educators’ retrospective evaluations of their preservice training, particularly in relation to their instructional mentors, and their extant self-efficacy. Educators rendered high appraisals of their self-efficacy during the pandemic, thereby attesting to their resilience in a convoluted epoch marked by profound uncertainty and characterized by exigent personal and professional adaptations [3]. 

**Figure 1:**
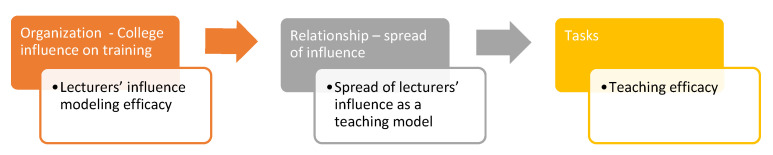
Teachers’ self-efficacy—chain of influence.

These findings led to the creation of a model depicting the chain of influences that, in our opinion, created the teachers’ high perceptions of their own competence (Figure 1). 

Likewise, the picture of the connections emerges as follows: The teachers’ retrospective perceptions of their preservice training indicate that the more they appreciate their training as positive and the more they value the lecturers, the greater their sense of self-efficacy. Seniority and marital status emerged as variables that also positively influence the sense of self-efficacy. The perception of self-efficacy develops with time and the experience of seniority. The more educated the teachers, the greater their sense of self-efficacy. The current study shows that the factors influencing teachers’ high sense of self-efficacy are good training from good lecturers appreciated by their students, as well as seniority, age, marital status, and higher education (master’s degree and CPD). 

**Figure 2:**
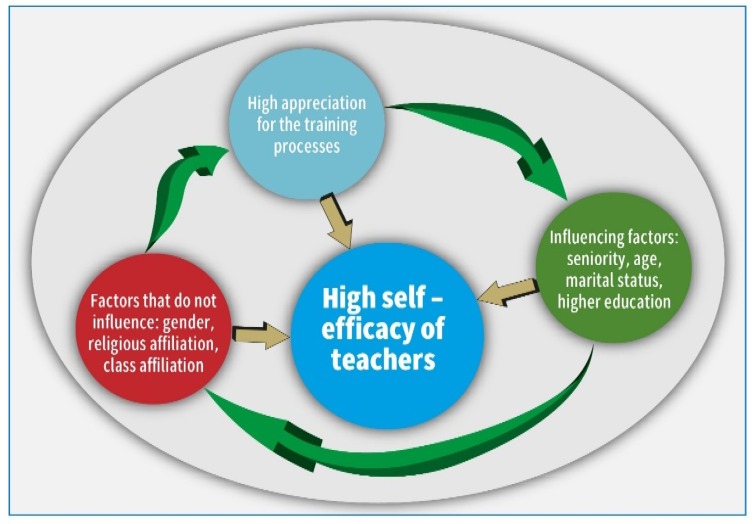
Factors affecting teachers’ sense of self-efficacy during the pandemic.

On the other hand, the variables of gender and socioeconomic status had no effect on the self-efficacy of the teachers sampled. Figure 2 encapsulates these insights. 

This highlights the crucial link between positive preservice training experiences and teachers’ SE during the COVID-19 pandemic. The alignment between the research questions, the findings, and the chain-of-influence model in Figure 1 underscores the significance of effective training and mentorship in fostering elevated self-efficacy among educators.

## 3. Summary of Discussion and Conclusions

This study, conducted amid the prolonged closure of the education system in Israel due to the COVID-19 pandemic, sheds light on crucial aspects of teacher self-efficacy and preservice training. Several research findings support and enrich the study’s implications.

Shifting to Remote Formats: Donitsa-Schmidt & Ramot’s (2022) [1] research provides context for the study, emphasizing the unprecedented challenges faced by educators in Israel during the pandemic that required a shift to remote pedagogical activities.

Positive Training Experiences and Mentorship: The study underscores the importance of positive training experiences and effective mentorship, aligning with existing literature on the significance of supportive training environments [42] (Darling-Hammond, 2017).

Influence of Effective Lecturers: The study highlights the influence of effective lecturers in shaping proficient educators, supporting findings from previous studies emphasizing the pivotal role of educators in cultivating adaptive capacities [42].

Three Dimensions of Professional Efficacy: Friedman and Kass’s (2000) [43] three dimensions of professional efficacy in Israel (task, relationship, and organizational aspects) resonate with the study’s identification of these dimensions, especially during the pandemic.

Teacher–Student Interactions and Academic Achievements:

Ayllón et al.’s (2019) [39] insights on the salient role of teacher–student interactions and their correlation with academic achievements align with the study’s emphasis on enduring impacts of such interactions on educators’ self-efficacy.

Role of Self-Reflection: The study’s alignment with Narayanan et al. (2022) [44], who emphasize the role of self-reflection in supporting teachers, contributes to the broader discourse on strategies for enhancing teacher resilience and effectiveness.

Global Relevance and Universality:

The study acknowledges its universal applicability, drawing parallels with shared challenges during the pandemic. This aligns with the broader literature emphasizing the global impact of crises on education and the need for adaptive teacher education programs (e.g., UNESCO, 2020). The study’s emphasis on the enduring impacts of teacher–student interactions contributes to the broader discourse on the role of self-efficacy in teachers’ professionalism [45] and reinforces the significance of teacher–student relationships.

In conclusion, this research establishes a significant connection between positive preservice training experiences and educators’ resilience during crises. The findings hold implications for global education and teacher education programs, emphasizing the importance of quality training, effective mentoring, and resilience-building strategies. Although the study was conducted in Israel, its insights are universally applicable due to shared challenges during the pandemic. Policymakers, educators, and institutions worldwide can leverage these findings to enhance teacher education programs, fostering resilience and adaptive capacities in the face of unforeseen circumstances. The study contributes to the broader discourse on the role of self-efficacy in teacher professionalism [46] but also highlights the enduring impacts of teacher–student interactions, making it relevant beyond scholarly circles and contributing to the broader goals of global education.

### Research Limitations

The questionnaire was sent to teachers who had completed their studies in the previous decade. Retrospective perceptions are subjective and rely on memories. On the other hand, the impact of lecturers who are remembered indicates a significant impact.

## Figures and Tables

**Table 1:** Characteristics, mean, SD, and validity of statements regarding lecturer perceptions and general self-efficacy (N = 165).

Measure/Statement	# of Statements	Min.	Max.	M	SD	α
To what extent did the lecturers present the curriculum and the perception criteria clearly?	1	1.00	5.00	4.05	0.96	--
To what extent did the supporting materials the lecturers gave me help with my studies?	1	1.00	5.00	3.72	1.05	--
Training structure	2	1.00	5.00	3.89	0.92	0.812
To what extent do I feel that I learned from the lecturers?	1	1.00	5.00	3.94	0.97	--
To what extent did the perception methods enable me to display my knowledge?	1	1.00	5.00	3.83	0.97	--
My perceptions of my self-efficacy	2	1.00	5.00	3.89	0.88	0.791
To what extent did I receive autonomous support from the lecturers?	1	1.00	5.00	3.75	1.13	--
To what extent did the lecturers’ involvement help me?	1	1.00	5.00	4.04	1.05	--
General perceptions of the lecturers’ contribution to my training	1	1.00	5.00	3.94	1.00	--
**Statement**	**Min**.	**Max.**	**M**			**SD**
I can achieve most of my educational goals (achievement orientation).	2	5	4.28			0.64
When faced with tough educational tasks I am sure I can carry them out (endurance).	2	5	4.28			0.67
In general, I think I can achieve what is important to me in teaching (task orientation).	2	5	4.29			0.69
I can succeed in any educational task when I am determined to do so (determination).	3	5	4.40			0.63
I can deal successfully with many educational challenges (achievement orientation).	2	5	4.37			0.64
I can carry out most of my educational tasks well (task orientation).	3	5	4.36			0.63
Even when things are tough, I can carry out my educational work well (endurance).	2	5	4.19			0.70
I’m confident I can complete most of my educational tasks (confidence).	3	5	4.37			0.63

**Table 2:** Distribution of demographic variables for research participants (N = 165).

	N	%	Min.	Max.	Mean	SD
Gender						
Female	115	69.7				
Male	50	30.3				
Age			25	60	37.96	8.04
Seniority			0	31	7.98	6.59
Family status						
Single	21	12.7				
Married	134	81.2				
In a relationship	4	2.4				
Divorced	5	3.0				
Widowed	1	0.6				
Education						
Teaching certificate	2	1.2				
B.A./B.Ed.	99	60.0				
M.A.	64	38.8				
Religion						
Jewish	120	72.7				
Muslim	19	11.5				
Christian	2	1.2				
Other	24	14.5				
SES						
Low	10	6.1				
Average	139	84.2				
High	16	9.7				

**Table 3:** Pearson coefficients between overall self-efficacy statements and lecturer evaluation measures (N = 165).

Statement		Self-Efficacy	Autonomy Support	Lecturer Involvement	HGeneral Evaluation
I can achieve my educational goals.	0.281 **	0.198 *	0.179 *	0.227 **	0.201 *
When faced with educational tasks I am sure I can carry them out.	0.358 **	0.285 **	0.298 **	0.302 **	0.296 **
I think I can achieve what is important to me in teaching.	0.270 **	0.213 **	0.267 **	0.101	0.169 *
I can succeed in any educational task when I am determined to do so.	0.238 **	0.197 *	0.188 *	0.122	0.203 **
I can deal successfully with many educational challenges.	0.244 **	0.184 *	0.234 **	0.155 *	0.156 *
I can carry out most of my educational tasks well.	0.204 **	0.129	0.149	0.149	0.123
Even when things are tough, I can carry out my educational work well.	0.288 **	0.185 *	0.283 **	0.180 *	0.171 *
I can complete most of my educational tasks.	0.247 **	0.186 *	0.235 **	0.215 **	0.148

* *p* < 0.005, ** *p* < 0.01.

**Table 4:** Lecturer evaluation and overall self-efficacy—men and women and *t*-test results (N = 165).

	Men (N = 50)		Women (N = 115)		
Lecturer Evaluation	M	SD	M	SD	t
Structure	4.03	0.85	3.83	0.95	1.31
Self-efficacy	3.94	0.92	3.87	0.86	0.50
Autonomy support	3.84	1.11	3.72	1.15	0.64
Lecturer involvement	4.22	1.00	3.96	1.06	1.44
Overall evaluation	4.08	0.94	3.88	1.02	1.20
	Men (N = 50)		Women (N = 115)		
Overall Self-Efficacy	M	SD	M	SD	t
I can achieve my educational goals.	4.20	0.68	4.31	0.62	1.00
When faced with educational tasks I am sure I can carry them out.	4.37	0.76	4.25	0.63	1.06
I think I can achieve what is important to me in teaching.	4.27	0.78	4.30	0.65	0.30
I can succeed in any educational task when I am determined to do so.	4.39	0.67	4.40	0.62	0.10
I can deal successfully with many educational challenges.	4.40	0.71	4.37	0.61	0.28
I can carry out most of my educational tasks well.	4.40	0.68	4.35	0.61	0.52
Even when things are tough, I can carry out my educational work well.	4.26	0.74	4.16	0.68	0.82
I can complete most of my educational tasks.	4.42	0.65	4.35	0.62	0.64

**Table 5:** Pearson correlations between age and seniority and statements of overall self-efficacy and lecturer evaluation indices (N = 165).

Lecturer Evaluation	Age	Seniority
Structure	0.139	0.070
Self-efficacy	0.168 *	0.091
Autonomy support	0.170 *	0.077
Lecturer involvement	0.092	0.016
Overall evaluation	0.083	−0.007
Overall Self-Efficacy	Age	Seniority
I can achieve my educational goals.	−0.006	0.087
When faced with educational tasks I am sure I can carry them out.	0.107	0.168 *
I think I can achieve what is important to me in teaching.	0.131	0.228 **
I can succeed in any educational task when I am determined to do so.	0.181 *	0.142
I can deal successfully with many educational challenges.	0.150	0.175 *
I can carry out most of my educational tasks well.	0.062	0.032
Even when things are tough, I can carry out my educational work well.	0.070	0.183 *
I can complete most of my educational tasks.	0.149	0.193 *

* *p* < 0.05, ** *p* < 0.01.

**Table 6:** Lecturer evaluation and overall self-efficacy according to level of education and *t*-test results (N = 165).

	Bachelor’s Degree (N = 101)		Master’s Degree (N = 64)		
Lecturer Evaluation	M	SD	M	SD	t
Structure	3.74	0.94	4.13	0.84	2.68 **
Self-efficacy	3.79	0.89	4.04	0.85	1.77
Autonomy support	3.55	1.15	4.08	1.04	3.00 **
Lecturer involvement	3.86	1.01	4.33	1.05	2.87 **
Overall evaluation	3.84	0.94	4.10	1.09	1.59
	Bachelor’s degree (N = 101)		Master’s degree (N = 64)		
Overall Self-Efficacy	M	SD	M	SD	t
I can achieve my educational goals.	4.21	0.69	4.38	0.52	1.64
When faced with educational tasks I am sure I can carry them out.	4.20	0.72	4.41	0.56	1.99 *
I think I can achieve what is important to me in teaching.	4.17	0.75	4.48	0.54	2.86 **
I can succeed in any educational task when I am determined to do so.	4.32	0.65	4.52	0.59	1.93
I can deal successfully with many educational challenges.	4.24	0.67	4.59	0.53	3.49 **
I can carry out most of my educational tasks well.	4.26	0.65	4.53	0.56	2.74 **
Even when things are tough, I can carry out my educational work well.	4.05	0.73	4.40	0.58	3.16 **
I can complete most of my educational tasks.	4.24	0.67	4.57	0.50	3.39 **

* *p* < 0.05, ** *p* < 0.01.

**Table 7:** Lecturer evaluation and overall self-efficacy based on family status and *t*-test results (N = 165).

	Single (N = 31)		Married (N = 134)		
Lecturer evaluation	M	SD	M	SD	t
Structure	3.71	0.99	3.93	0.90	1.20
Self-efficacy	3.82	0.99	3.90	0.86	0.46
Autonomy support	3.58	1.39	3.80	1.07	0.95
Lecturer involvement	4.10	1.16	4.03	1.02	0.32
Overall evaluation	4.03	1.02	3.92	1.00	0.58
	Single (N = 31)		Married (N = 134)		
Overall Self-Efficacy	M	SD	M	SD	t
I can achieve my educational goals.	4.03	0.80	4.34	0.58	2.45 *
When faced with educational tasks I am sure I can carry them out.	4.06	0.63	4.33	0.67	2.03 *
I think I can achieve what is important to me in teaching.	4.19	0.75	4.31	0.68	0.86
I can succeed in any educational task when I am determined to do so.	4.23	0.73	4.43	0.61	1.56
I can deal successfully with many educational challenges.	4.32	0.70	4.39	0.63	0.50
I can carry out most of my educational tasks well.	4.23	0.67	4.40	0.62	1.37
Even when things are tough, I can carry out my educational work well.	4.03	0.80	4.22	0.67	1.36
I can complete most of my educational tasks.	4.23	0.67	4.40	0.62	1.41

* *p* < 0.05.

## Data Availability

The data presented in this study are available on request from the corresponding author.
